# Are orexin antagonists capable of improving both insomnia and vasomotor symptoms in menopausal women?

**DOI:** 10.1017/S109285292510076X

**Published:** 2025-11-27

**Authors:** Christine E. Dri, Yang Jing Zheng, Roger S. McIntyre

**Affiliations:** 1 Brain and Cognition Discovery Foundation, Canada; 2 Institute of Medical Science, University of Toronto, Canada; 3 Department of Psychiatry, University of Toronto, Canada; 4 Department of Pharmacology and Toxicology, University of Toronto, Canada

**Keywords:** Orexin, menopause, dual orexin receptor antagonist, DORA, suvorexant, lemborexant, midlife, vasomotor symptoms, VMS, daridorexant, seltorexant, hormone replacement therapy

## Abstract

The menopausal period in women is characterized by neuroendocrine alterations, which is in part mediated by the reduction in circulating estrogen. During this transition, many perimenopausal and menopausal women experience sleep disturbances and increased susceptibility to sleep-related disorders. Sleep disruptions are partially attributed to nighttime vasomotor symptoms (VMS), which exacerbates the insomnia risk in the menopausal woman. Converging data implicate the orexin system in the pathophysiology of insomnia and VMS, particularly through regulation of arousal, thermoregulation, and sympathetic outputs. Estrogen decline due to menopause is postulated to modulate orexin signaling, thereby heightening sympathetic drive and thermoregulatory instability. Given this potential mechanistic framework, orexin receptor antagonists, notably dual orexin receptor antagonists (DORAs), have been proposed as alternative menopausal therapeutics. Herein, we aim to examine preclinical, translation, and clinical literature assessing the therapeutic potentials of DORAs as a nonhormonal intervention for the mitigation of insomnia and VMS in midlife women.

Menopause is a period marked by profound physiological and psychological alterations in women largely mediated by a reduction in hormonal levels, with diminished estrogen production being a pronounced change. During this transition, both perimenopausal and menopausal women often report symptoms related to sleep disorders, including but not limited to, difficulty initiating and maintaining sleep, reduction in sleep quality, as well as increased in incident sleep-related disorders (eg, obstructive sleep apnea).^1^ For example, sleep disturbances are reported by 16–47% of perimenopausal women and 35–60% of postmenopausal women, in contrast to approximately 5% reported by premenopausal women.^1^ Sleep disturbances are associated with a diminished quality of life, heightened cardiovascular and metabolic disease risks, sleep apnea, weight gain and obesity.^2^ ^–^^6^

Both sleep apnea and sleep disorders become more prominent with increased age, a pattern that is exacerbated during the menopausal transition.^6^ Sleep disruption meaningfully and predictably disrupts quality of life and increases the risk for mental health disorders, including major depressive disorder (MDD), anxiety, and suicidality in women during menopausal transition.^3^ ^,^^4^ ^,^^7^ ^,^^8^ Moreover, alterations in sleep are known to be significantly associated with more difficult-to-treat mental disorders (eg, treatment resistant depression).^9^

Sleep disturbances are partially attributed to vasomotor symptoms (VMS) in menopausal women. According to the Study of Women’s Health Across the Nations, VMS, characterized by hot flashes and night sweats, is reported to affect 50–72% of menopausal women in the United States.^10^ VMSs are also the most common menopausal symptoms identified as a chief complaint during the clinical encounter with healthcare professionals.^11^ VMS related to menopause have also been associated with insomnia-like symptoms, including nighttime awakenings further exacerbating the insomnia risk in menopausal woman.^12^ For example, it is reported that when mimicking menopause in healthy women, they report a significant increase in wakefulness after sleep onset (WASO) and nighttime awakenings, as confirmed by patient-reported sleep diaries and at-home polysomnography studies that coincide with VMS.^13^ Separately, objective measures of VMS in menopausal women were reported to be associated with nighttime awakenings in 78% of respondents.^13^

The menopausal transition is marked by a decrease in sex hormones, specifically a reduction of estradiol, which is a known inhibitor of orexin production.^15^ For example, estrogen’s modulatory effect on orexin synthesis and release is instantiated by normalization of circulating orexin levels subsequent to estrogen administration.^16^ Orexin, a neuropeptide produced in the perifornical and lateral hypothalamic region, plays a critical role not only in wakefulness and arousal but also in inflammatory response, metabolic homeostasis, reward/stress response, cognitive function and sympathetic output (Table 1).^8^ ^,^^17^ It is hypothesized that the significant increase in plasma orexin levels in menopausal women, contemporaneous with a decrease in circulating estrogen results in altered orexin-mediated sympathetic drive and thermoregulation, resulting in not only an increased risk for insomnia and VMS but also VMS exacerbation of insomnia (Figure 1). ^18^ Hormone replacement therapy (HRT) is commonly prescribed as first-line treatment for menopausal women with VMS. A recent meta-analysis reported that estrogen replacement therapy reduced VMS by approximately 75% and severity by 83%.^19^ Although HRTs can temporarily elevate estrogen levels, repeated administration has limited long-term and sustained effects, particularly for improvements in sleep quality, body weight, and overall wellbeing.^16^ Additionally, despite revised estimates of the risk/benefit of HRT with respect to vascular and/or cancer-related hazard, a demand for nonhormonal treatment approaches for menopausal-related symptoms is apparent, resulting in the frequent utilization of over-the-counter agents, prescribed off-label agents (eg, selective serotonin reuptake inhibitors) as well as nonpharmacologic approaches (eg, cognitive behavioral therapy) to treat menopausal-related symptoms, notably VMS.^20^ ^,^^21^

**Table 1.** Rationale for the Use of Orexins to Treat VMS-Related Insomnia Symptoms in Menopausal Women

Rationale for the use of orexin
Orexin is a neuropeptide that is synthesized in the perifornical and lateral hypothalamic region Orexinergic neurons project to brain regions (eg, noradrenergic locus coeruleus) involved in arousal and wakefulness Orexin regulates the following responses: wakefulness, arousal, inflammatory response, metabolic homeostasis, reward/stress response, cognitive function, and sympathetic output^14^ Orexin plays a role in sympathetic control by interacting with various brain regions such as the nucleus of the solitary tract (NTS) and the rostral ventrolateral medulla^14^ Antagonism of the orexin system could potentially improve sleep and sympathetic dysregulation to alleviate VMS-related insomnia symptoms in menopausal women

**Figure 1.**
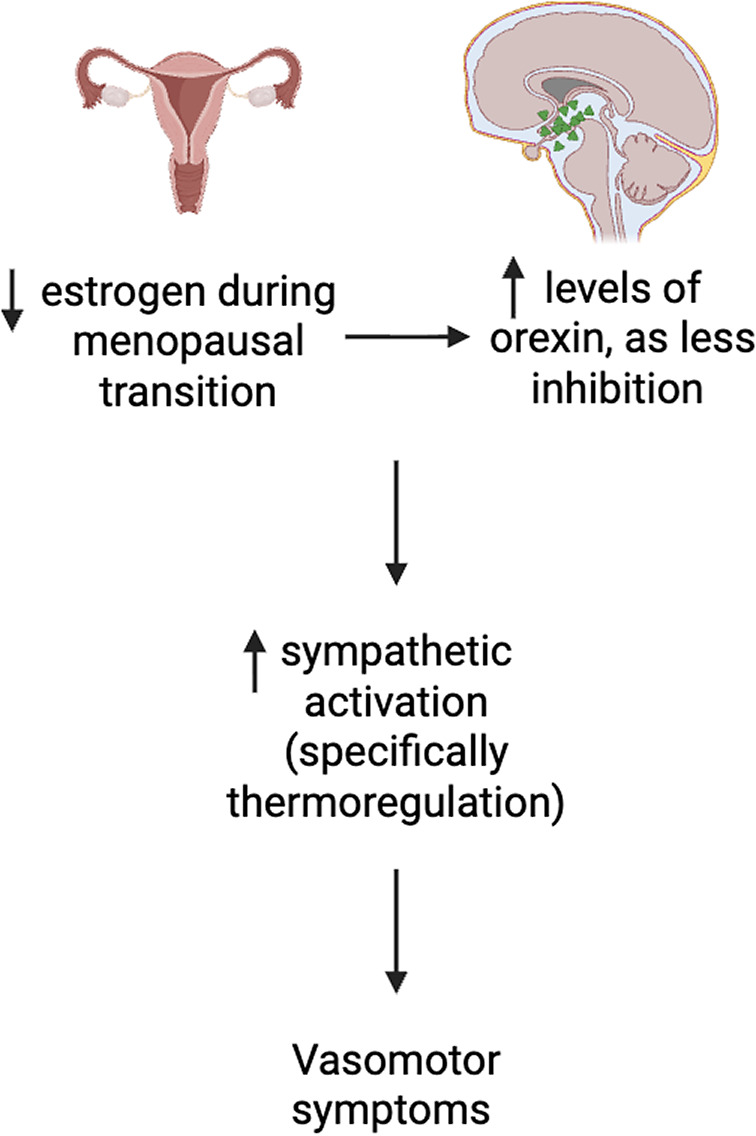
Proposed mechanistic pathway between estrogen, orexin and vasomotor symptoms in menopausal women. Created using BioRender.

Preliminary results from preclinical pharmacologic and clinical studies suggest that the dual orexin receptor antagonists (DORAs), which are FDA-approved for the treatment of insomnia (ie, suvorexant, lemborexant, and dardiorexant), may also be preferred therapeutics in the treatment of menopausal-related insomnia. In addition, DORAs may also have therapeutic potential in mitigating VMS-related insomnia-like symptoms and provide an alternative therapeutic strategy to HRT for menopausal women. Herein, we aim to briefly synthesize the extant and preliminary literature documenting the mechanistic rationale and potential therapeutic utility of DORAs in the simultaneous treatment of insomnia and VMS in midlife women.

Orexin antagonism has been proposed to exert mitigative effects on sleep disorders and sympathetic dysregulation (eg, thermoregulatory reflexes) by targeting the orexin system, wherein the DORAs competitively and reversibly binds to orexin-associated receptors subserving arousal systems (Table 1).^22^ The aforementioned mechanism is postulated to dampen the activity of endogenous orexin, consequently reducing maladaptive arousal, reducing latency to sleep and WASO, as well as reducing sympathomimetically mediated cardiovascular tone and thermoregulatory homeostasis disturbance, among others.^14^ ^,^^17^ ^,^^23^ It can be hypothesized that the aforementioned neurophysiologic effects provide the rationale for the implementation of DORAs, specifically in women during midlife experiencing insomnia and VMS.

Convergent evidence provides preliminary support for the testable and viable hypothesis that orexin antagonism reduces the frequency and severity of VMS in association with insomnia disorder in menopausal animal models and midlife women. For example, Federici et al.^24^ reported in an ovariectomy (OVEX) mouse model that the loss of estrogen resulted in hyperactivity of the orexin system. The OVEX mouse model surgically induces a menopausal state mirroring associated symptoms reported in peri- and postmenopausal women. Exposure to an anxiogenic stimulus (ie, FG-7142) elicited heightened vasomotor responses, as demonstrated via 6–7 °C increase in tail skin temperature.^24^ Whereas selective orexin receptor type 1 (OX1R) and orexin receptor type 2 (OX2R) agonists partially attenuated tail temperature, DORA administration completely blocked the vasomotor response.^24^ In addition to thermoregulatory changes, the aforementioned study also reported that stress and anxiety measures were significantly increased in severity and frequency, coinciding with increased vasomotor response in the OVEX mice.

Substantiating the preclinical findings, two clinical studies were identified that reported on the efficacy of orexin antagonists (ie, suvorexant and lemborexant) in the treatment of insomnia and/or VMS in peri- or postmenopausal women.^18^ ^,^^25^ Terauchi et al.^25^ conducted a post hoc analysis of a 12-month, randomized, double-blind, placebo-controlled trial that examined the efficacy and safety of lemborexant (LEM; 5 and 10 mg) in midlife women (N = 280; 40–58 years) with insomnia disorder. The aforementioned study assessed sleep and fatigue outcomes, including WASO, sleep onset latency (SOL), sleep efficiency (SE), total sleep time (TST), Insomnia Severity Index (ISI), Fatigue Severity Scale (FSS), and Patient Global Impression–Insomnia.^25^

At 6 months, 10 mg LEM (0.74 [95% CI, 0.57 to 0.97], P = 0.031) significantly reduced SOL compared to placebo, whereas 5 mg LEM exhibited a numerical but nonsignificant improvement in SOL scores.^25^ Both LEM doses yielded a higher SE (10 mg LEM +17.2%; 5 mg LEM +15.9%) than placebo (placebo +12.5%). Likewise, longer TST (10 mg LEM +90.6 minutes, 5 mg LEM +80.6 minutes, placebo +59.9 minutes) and reduced WASO (10 mg LEM −54.5 minutes, 5 mg LEM −50.1 minutes, placebo −37.0 minutes) were observed.^25^ Mean ISI and FSS both decreased in treatment (ISI −9.8 to −10.7; FSS −9.0 to −9.9) compared to placebo (ISI −8.3; FSS −7.4), with sustained benefits throughout the 12-month study period.^25^ Overall, LEM was relatively well tolerated, with mild to moderate adverse events, implicating orexin antagonism as a potential therapeutic for midlife women with insomnia disorder.

Separately, Rahman et al.^18^ conducted a 4-week randomized double-blinded study assessing the effect of suvorexant on insomnia and VMS in midlife women (N = 57; mean age 54.3 ± 4.3 years). Participants were randomized to receive either suvorexant (n = 27; 10 or 20 mg) or placebo (n = 29). Sleep-related outcome measures were ISI, WASO, SOL, TST, and SE scores.^18^ VMS frequency was also recorded (twice daily; daytime and nighttime), while exploratory outcomes included the Hot Flash-Related Daily Interference Scale for impairment, the Patient Health Questionnaire-9 (PHQ-9) to evaluate depressive symptoms, and the Menopause-Specific Quality-of-Life scale.^18^

A total of 53 women completed the study and were included in the full analysis set for statistical comparison.^18^ Rahman et al. reported that suvorexant administration was associated with a significant within-person reduction in insomnia symptom severity, with a greater ISI score reduction with treatment (−8.1 [95% CI, −10.2 to −6.0]) compared to placebo (−5.6 [95% CI, −7.4 to −3.9], P = 0.04).^18^ Suvorexant also significantly reduced nighttime self-reported VMS frequency (P < 0.01).^18^ In addition, suvorexant treatment was associated with a significant reduction in nighttime, but not daytime, VMS. Taken together, these findings provide empirical support for the possibility that a DORA may not only be effective and well-tolerated in the treatment of insomnia but also VMS in midlife women.^17^ ^,^^22^ ^,^^23^ ^,^^26^

An interim summary of the modest evidence base in this area indicates that DORAs represent a promising therapeutic research approach that is effective, mechanistically rational and supported by a convergence of preclinical and clinical studies. Furthermore, DORAs are noted to be well-tolerated and safe, with recent pharmacovigilance analyses and controlled trials suggesting that these agents have a relatively safe psychiatric profile.^27^ ^,^^28^ Given limited clinical evidence, larger, randomized controlled clinical trials in menopausal women are needed to provide information regarding the long-term safety and extended efficacy of DORAs for VMS-associated insomnia symptoms. In addition, whether single orexin receptor antagonists, such as seltorexant which is currently in late phase III development for the adjunctive treatment of MDD, is also able to benefit VMS would be of interest to the field.^29^ ^,^^30^
